# Novel RNA-binding properties of the MTG chromatin regulatory proteins

**DOI:** 10.1186/1471-2199-9-93

**Published:** 2008-10-24

**Authors:** Stefano Rossetti, Leontine van Unen, Nicoletta Sacchi, Andre T Hoogeveen

**Affiliations:** 1 Cancer Genetics Program, Roswell Park Cancer Institute, Elm and Carlton Streets, Buffalo, NY 14263, USA; 2 Department of Clinical Genetics, Erasmus MC, Dr Molewaterplein 50, 3015GE Rotterdam, The Netherlands

## Abstract

**Background:**

The myeloid translocation gene (MTG) proteins are non-DNA-binding transcriptional regulators capable of interacting with chromatin modifying proteins. As a consequence of leukemia-associated chromosomal translocations, two of the MTG proteins, MTG8 and MTG16, are fused to the DNA-binding domain of AML1, a transcriptional activator crucial for hematopoiesis. The AML1-MTG fusion proteins, as the wild type MTGs, display four conserved homology regions (NHR1-4) related to the *Drosophila *nervy protein. Structural protein analyses led us to test the hypothesis that specific MTG domains may mediate RNA binding.

**Results:**

By using an RNA-binding assay based on synthetic RNA homopolymers and a panel of MTG deletion mutants, here we show that all the MTG proteins can bind RNA. The RNA-binding properties can be traced to two regions: the Zinc finger domains in the NHR4, which mediate Zinc-dependent RNA binding, and a novel short basic region (SBR) upstream of the NHR2, which mediates Zinc-independent RNA binding. The two AML1-MTG fusion proteins, retaining both the Zinc fingers domains and the SBR, also display RNA-binding properties.

**Conclusion:**

Evidence has been accumulating that RNA plays a role in transcriptional control. Both wild type MTGs and chimeric AML1-MTG proteins display *in vitro *RNA-binding properties, thus opening new perspectives on the possible involvement of an RNA component in MTG-mediated chromatin regulation.

## Background

The myeloid translocation gene (MTG) protein family includes three human members: MTG8 (ETO/CBFA2T1) [1-3], MTGR1 (CBFA2T2) [4-6] and MTG16 (CBFA2T3) [7]. The MTG proteins share four conserved domains that can be traced to the *Drosophila *protein nervy, and therefore called nervy homology regions (NHR1-4) [6]. These domains carry information for distinct, but integrated, functional properties. The NHR1 domain can positively or negatively modulate transcription through interaction with either co-repressors or transcriptional activators [8]. The NHR2 domain is required for interaction with other MTG proteins and with the transcriptional co-repressor Sin3A [6,9-11]. The NHR4 domain, even if it contains two zinc finger (ZF) domains, does not mediate DNA-binding [12,13]; instead, it binds both co-repressor proteins, including N-CoR/SMRT, and histone deacetylases (HDACs) [11,14,15]. We and others showed that the MTG proteins can act as chromatin repressors due to their ability to recruit HDAC activity, either directly [10,11,16,17] or via the co-repressors N-CoR/SMRT and Sin3A [14,15,18]. Further, it has been demonstrated that both MTG8 and MTG16 can induce transcriptional repression of reporter genes [10,15,17,19]. Since the MTG proteins do not bind to DNA directly, their transcriptional repressive action is dependent on the binding to transcription factors able to recognize specific target-genes ([20] and references within).

As a consequence of the leukemia-associated chromosomal translocations t(8;21) and t(16;21), MTG8 and MTG16 are fused to AML1 (RUNX1), a transcription factor crucial for hematopoiesis. The resulting fusion proteins AML1-MTG8 (AML1-ETO) and AML1-MTG16 retain the DNA-binding domain of AML1 (Runt Homology Domain, RHD) and all the four functional NHR domains of the MTG proteins (for detailed reviews see [12,21-24]). Both AML1-MTG8 and AML1-MTG16 can bind to AML1-target genes, recruit HDAC activity, and induce a repressed chromatin state [20,25-27]. *In vitro *studies suggest that epigenetic downregulation/silencing of these target genes may be a key step in leukemogenesis [12,21-24].

More and more evidence has been accumulating that RNA, in particular non-coding RNA (ncRNA), can play an important role in the epigenetic control of chromatin [28-30]. The MTG proteins are transcriptional regulators equipped with non-DNA-binding ZF domains, which have been described to mediate protein-RNA interactions in other proteins [31]. Based on this observation, we previously hypothesized that transcriptional regulation by the MTG proteins might involve an RNA component [20]. To start to address this hypothesis, we set out to investigate whether the MTG proteins have RNA-binding properties. By using a well established RNA-binding assay based on synthetic RNA homopolymers [32], here we show that indeed this is the case. Two regions mediate the RNA binding: the zinc-finger domains in the NHR4 region and a novel RNA-binding short basic region (SBR) proximal to the NHR2 region. We further show that the two oncogenic fusion proteins AML1-MTG8 and AML1-MTG16, retaining these two regions, maintain also RNA-binding properties.

## Results

### The MTG proteins have RNA-binding properties

**Figure 1:**
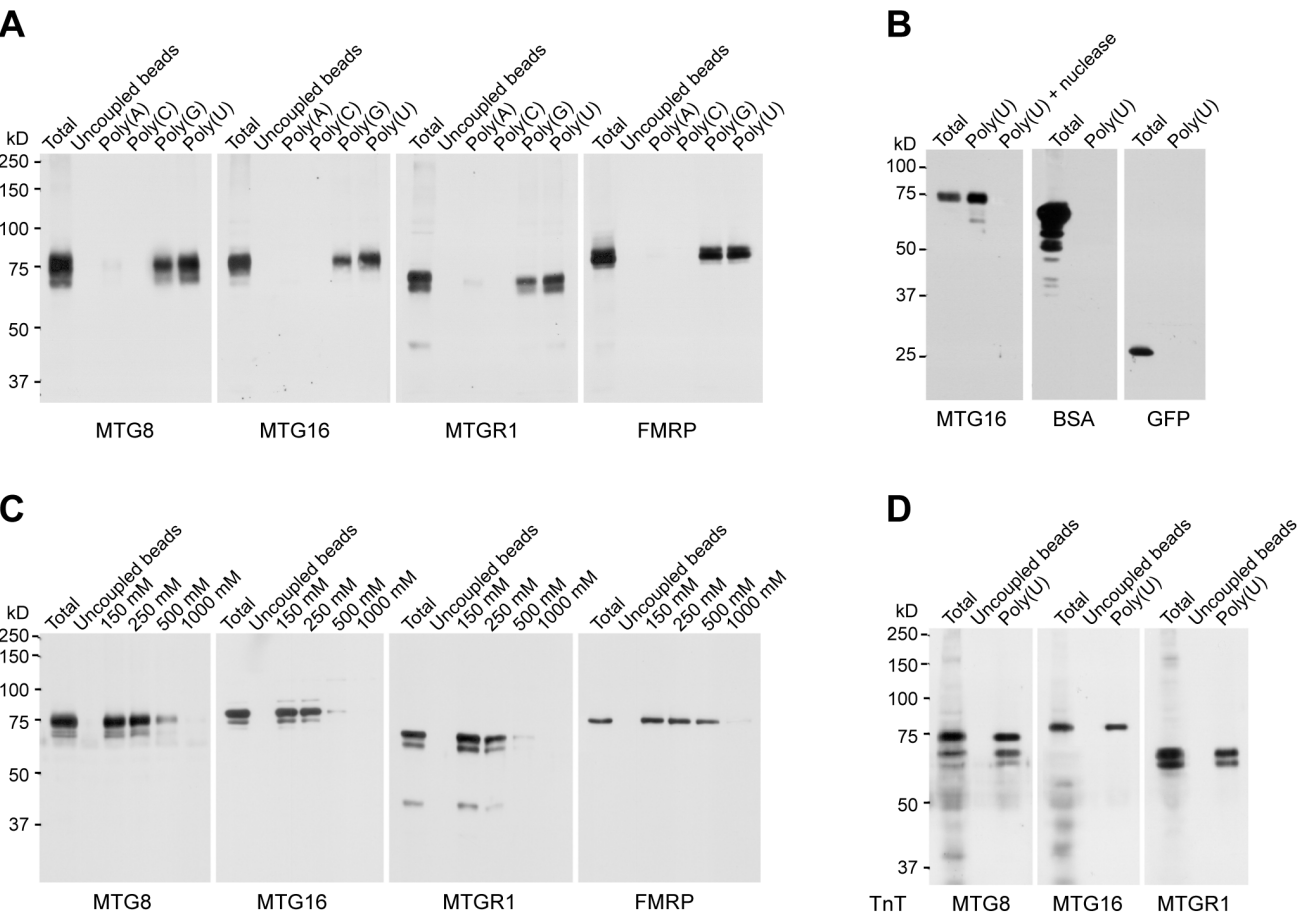
**The MTG proteins have RNA-binding properties**. **A**. RNA-binding assay using Sepharose-conjugated RNA homopolymers followed by Western Blotting shows that MTG8, MTG16 and MTGR1 exogenously expressed in COS cells bind to poly(G) and poly(U), while do not bind to poly(A), poly(C) and uncoupled Sepharose beads. FMRP, which we used as a positive control, shows similar RNA-binding properties. **B**. Binding specificity is shown both by the MTG inability to bind poly(U) after digestion with micrococcal nuclease (shown here for MTG16), and by the inability to bind poly(U) of two non-RNA-binding proteins, BSA (10 μg) and GFP (transiently expressed in COS cells). **C**. Poly(U)-binding at different concentrations of NaCl shows the strength of RNA interaction of the MTG proteins and the control RNA-binding protein FMRP. **D) ***In vitro *transcribed and translated (TnT) MTG proteins maintain the ability to bind poly(U).

We investigated the RNA-binding properties of MTG8, MTG16 and MTGR1 by analyzing their ability to interact with four synthetic RNA homopolymers, poly(A), poly(C), poly(G) and poly(U), coupled to Sepharose beads. This method has been previously proven to be suitable for studying RNA-binding properties of RNA-binding proteins, including the Fragile × mental retardation protein FMRP, which we used in this study as a positive control [33-35]. The three MTGs, exogenously expressed in COS cells, display binding to both poly(U) and poly(G), but no binding to poly(A) and poly(C), thus showing the same properties of the control RNA-binding protein FMRP (Figure 1A). All MTGs did not bind uncoupled Sepharose beads, indicating specific affinity for RNA (Figure 1A). For the remainder of this study we chose to use only poly(U) RNA. Digestion with micrococcal nuclease of the Sepharose-conjugated poly(U) homopolymer apparently abolishes MTGs precipitation (here shown for MTG16), demonstrating that the binding occurs via poly(U) RNA (Figure 1B). In addition, we showed that known non-RNA-binding proteins, such as BSA and GFP, were not able to bind poly(U) RNA under the experimental conditions used (Figure 1B). These indicate that the RNA-binding properties of the MTG proteins are specific.

Next, we determined the strength of MTGs binding to poly(U) beads in the presence of 150, 250, 500 and 1000 mM NaCl. All MTGs bound the poly(U) homopolymer at the physiological salt concentration of 150 mM (Figure 1C). The binding was stable up to 250 mM NaCl, while it weakened at higher salt concentrations (Figure 1C), which is not uncommon for other RNA-binding proteins [36,37]. The RNA-binding strength of the MTG proteins is similar to the one reported for RNA-binding proteins that, like the MTGs [20], have multiple functions [38].

Finally, we investigated the influence of posttranslational modifications of the MTG proteins on the RNA binding. MTG proteins produced in an *in vitro *transcription/translation system, in which posttranslational modifications do not occur, retained the ability to interact with poly(U) RNA (Figure 1D). This indicates that posttranslational modifications are not directly necessary for the observed RNA binding.

### Deletion of the Zinc-finger domains is not sufficient to abolish RNA-binding properties

**Figure 2:**
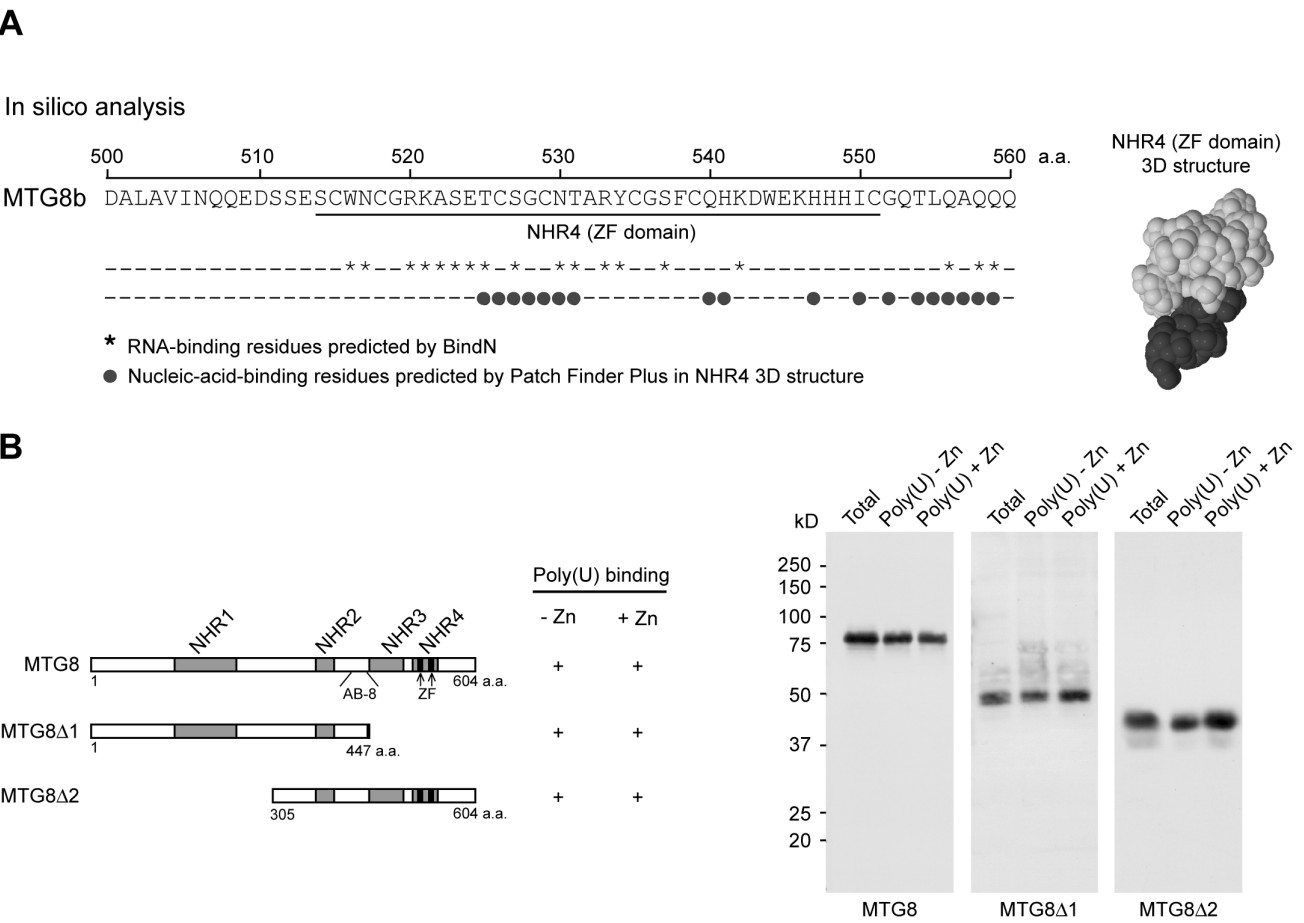
**Deletion of the Zinc finger domains in the NHR4 region is not sufficient to abolish RNA binding**. **A**. *In silico *analysis of the MTG8 NHR4 domain primary structure (left) and solution structure (right), performed by using BindN and Path Finder Plus, respectively, identifies putative RNA-binding residues (left). **B**. Scheme of the MTG8 deletion mutants showing MTGs conserved domains (NHRs), the zinc finger (ZF) domains and the epitope recognized by the anti-MTG8 antibody (AB-8) (left). Poly(U)-binding assay performed in the presence or the absence of ZnCl_2 _(50 μM) shows that neither deletion of MTG8 C-terminus (MTG8Δ1), containing the ZF domains, nor deletion of the N-terminus (MTG8Δ2) are sufficient to abrogate the binding (right).

The NHR4, a region conserved across all the MTGs, contains two Zinc Finger (ZF) domains [6]. ZF domains are known to have DNA-binding properties, but they have been described to mediate also interaction with RNA [31]. *In silico *analysis of the MTG8 NHR4 structure indeed suggests that this region is a putative RNA-binding domain. First, we analyzed the primary structure of the NHR4 region by using the BindN program [39]. This analysis predicted several RNA-binding residues between aminoacid 516 and 542 (Figure 2A, left). Further, we analyzed the MTG8 NHR4 solution structure, previously solved by nuclear magnetic resonance (NMR) spectroscopy [40] and deposited in the Protein Data Bank (PDB), by using Patch Finder Plus [41,42]. The Patch Finder Plus algorithm extracts from the three-dimensional (3D) protein structure positively charged electrostatic patches, which are known to mediate protein-nucleic-acid interactions. The algorithm predicted on the surface of the NHR4 domain a large positive patch (Figure 2A, right), whose amino acid residues partially overlap with the RNA-binding residues predicted by the analysis of the NHR4 primary structure (Figure 2A, left). Because the MTG proteins do not have DNA-binding properties [13], it is conceivable to hypothesize that this positive patch mediates protein-RNA binding. For this reason, we further tested the predicted RNA-binding *in vitro*, by developing two MTG8 deletion mutants either lacking the ZF-containing C-terminus (MTG8Δ1) or the N-terminus (MTG8Δ2) (Figure 2B, left). The MTG8 epitope recognized by our AB-8 antibody [16] (Figure 2B, left) allowed the detection of these deletion mutants without using protein tags, which might interfere with the RNA-binding. Deletion of the MTG8 C-terminus (MTG8Δ1) did not affect the binding to poly(U) (Figure 2B, right), thus indicating that RNA-binding domains other than the ZF might be present. This supposition is further supported by the observation that both MTG8 and MTG8Δ1 bind to poly(U) even in the absence of ZnCl_2 _(Figure 2B, right), which would be necessary for a ZF-mediated interaction. Since deletion of MTG8 N-terminus (MTG8Δ2) did not abolish Zinc-independent binding to poly(U) (Figure 2B, right), we hypothesized the presence of an additional RNA-binding domain common to the two deletion mutants, in the region encompassing a.a. 305–447.

### Identification of a Zinc-independent RNA-binding domain proximal to the NHR2 region

**Figure 3:**
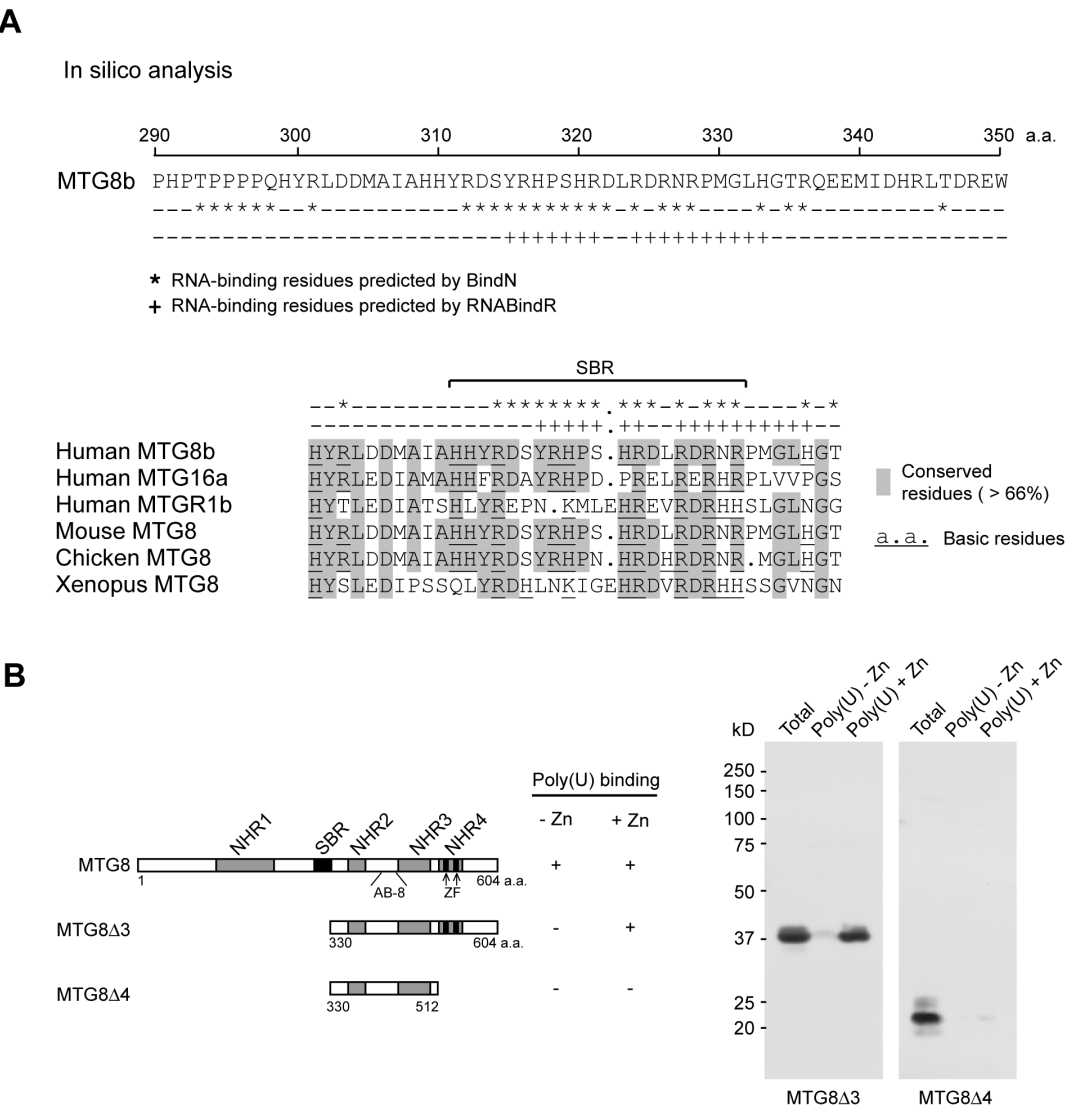
**Identification of SBR, a novel RNA-binding domains proximal to NHR2**. **A**. *In silico *analysis of the MTG8 protein sequence with BindN and RNAbindR software predicts RNA-binding residues in a region between a.a. 316 and a.a. 333 (top). This sequence is part of a short basic region (SBR) highly conserved across the three human MTGs and across different species (bottom). **B**. Deletion of MTG8 N-terminus, containing the SBR (MTG8Δ3), abrogates Zinc-independent poly(U)-binding; further deletion of MTG8 C-terminus, containing the Zinc Finger (ZF) domains (MTG8Δ4), abrogates also Zinc-dependent poly(U)-binding.

To search for potential RNA-binding domains other than the NHR4 in the MTG8 protein, we used, in addition to BindN, a second software, RNAbindR. RNAbindR is a computational tool able to predict RNA-binding amino acids from a protein primary sequence, based on interactions from structures of known protein-RNA complexes [43]. Both programs predicted an MTG8 region rich in RNA-binding residues between a.a. 310 and a.a. 333 (Figure 3A, top). Analysis of the MTG8 sequence from other species and other human MTG proteins identified a conserved short basic region (which we named SBR) within a.a. 309 and a.a. 328 (Figure 3A, bottom). This region has a high content in basic amino acids, which are often involved in nucleic acid binding [44]. Specifically, the arginines at position 312, 321, 324 and 326 and other basic residues at position 317 and 358 are conserved from Xenopus to Human (Figure 3A, bottom). Since the three-dimensional structure of the SBR is not known, we could not test for the presence of positively charged protein patches. However, we established the RNA-binding properties of the SBR *in vitro*. By deleting the SBR from MTG8Δ1, we obtained the deletion mutant MTG8Δ3, which lacks a.a. 1–329, but retains the ZF domains in the C-terminus (Figure 3B, left). MTG8Δ3 was able to bind poly(U) only in the presence of ZnCl_2 _(Figure 3B, right), indicating that the SBR is responsible for the Zinc-independent binding to RNA. Another deletion mutant, MTG8Δ4, missing both the zinc-finger domain and the N-terminal part, including the SBR, did not bind to poly(U) either in the presence or in the absence of ZnCl_2 _(Figure 3B, right), indicating that the ZF domains mediate the Zinc-dependent RNA-binding. In conclusion, two regions mediate MTG8 RNA-binding: the canonical ZF domains in the NHR4 region and a novel Zinc-independent RNA-binding domain corresponding to the SBR region.

### The fusion proteins AML1-MTG8 and AML1-MTG16 retain the RNA-binding properties of wild type MTGs

**Figure 4:**
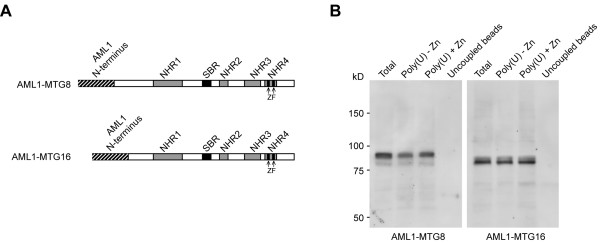
**AML1-MTGs fusion proteins maintain MTGs RNA-binding properties**. **A**. Both the ZF domains and the SBR region are present in AML1-MTG8 and AML1-MTG16. **B**. AML1-MTG8 and AML1-MTG16 are able to bind poly(U) both in the presence and the absence of ZnCl_2_.

As a consequence of the leukemia-associated chromosome translocations t(8;21) and t(16;21), almost the entire MTG8 and MTG16 protein moieties are fused to the RHD domain of AML1, leading to the chimeric AML1-MTG8 and AML1-MTG16 proteins, respectively [1-3]. These chimeric proteins retain the AML1 DNA-binding domain and all the MTGs functional domains, including the ZF and the SBR RNA-binding domains (Figure 4A). Here we show that both fusion proteins bind to poly(U) both in the presence and the absence of ZnCl_2 _(Figure 4B), thus indicating that the RNA-binding properties of the wild type MTG proteins are maintained in their chimeric counterparts.

## Discussion

The MTG proteins are transcriptional regulators capable of networking with their own family protein members and a variety of transcriptional regulatory proteins. Apparently, the major MTGs' action relies on the ability of these adaptor proteins to establish multiple interactions, on one hand with canonical DNA-binding transcription factors and, on the other hand, with chromatin regulatory proteins, including repressor proteins and histone modifying enzymes (reviewed in [20]). The MTGs carry out distinct, but integrated, functional interactions through conserved domains, the NHR1-4, homologous to the *Drosophila *protein Nervy. Despite the presence of two zinc finger (ZF) motifs in one of the domains (NHR4), the MTG proteins do not exert their transcriptional regulatory function by direct DNA binding [12,13].

Based on the observation that ZF motifs can mediate not only DNA-protein interactions, but also RNA-protein interactions [44], we previously hypothesized that the ZF-containing NHR4 domain could confer RNA-binding properties to both wild type and chimeric MTG proteins [20]. In this study, by using an *in vitro *assay based on RNA homopolymers binding [32], we show that the MTG proteins specifically bind to RNA. We unequivocally demonstrate the binding specificity by performing a series of experiments. First, we showed that non-RNA-binding proteins cannot be precipitated by RNA hompolymers under the same conditions used for the MTGs. Second, digestion of the poly(U) RNA homopolymer completely abolished the MTGs binding. Finally, the MTG-RNA interaction was abrogated by deletion of specific MTG domains. By combining *in silico *protein analyses and development of MTG deletion mutants, we found that the RNA binding is not only mediated by the ZF domains in the NHR4 region, but entails also a novel, Zinc-independent, RNA-binding region proximal to NHR2, the SBR region. The SBR domain seems to be highly conserved across the three human MTGs and the MTGs of different species, and it is rich in basic amino acid residues, a feature frequently observed in RNA binding domains [44]. To our knowledge, this region is a *bona fide *novel RNA binding domain, whose primary and secondary structures do not resemble canonical RNA-binding domains [44].

The aberrant AML1-MTG fusion proteins retain the ability of interplaying with both repressor proteins and/or histone modifying enzymes, and can induce an altered epigenetic status at the chromatin of both coding and non-coding AML1-target genes [26,27,45]. Apparently, the fusion of two MTGs, MTG8 and MTG16, to the RHD region of AML1 does not affect the *in vitro *RNA-binding properties.

Whether the wild type and chimeric MTG proteins bind RNA *in vivo *remains to be established. The MTG proteins, like the well-known RNA-binding protein FMRP [33], bind preferentially to poly(U) and poly(G). This might suggest a potential affinity for RNAs rich in U and/or G, such as mRNAs containing short tandem repeats (STR) made of GU dinucleotides [46] or long poly(U) stretches ([32] and references within) in their 3'untranslated region (UTR). Further, based on the strength of RNA binding, the MTG proteins might mediate transient protein-RNA interactions. It is noteworthy that transient RNA-protein interactions often characterize multifunctional proteins, such as chaperone proteins, with RNA-binding strengths similar to the ones displayed by the MTGs [38].

Interestingly, the two domains that we found to be involved in RNA binding in either a Zinc-dependent or a Zinc-independent fashion are also capable of interacting with specific chromatin regulatory proteins. Specifically, the ZF-containing NHR4 domain is known to interact with both the N-CoR/SMRT proteins and HDACs [10,11,14,15,18], while the SBR domain is overlapping with a region (aa. 300–343) involved in HDAC3 binding [11]. Whether an RNA component initiates, or contributes, to the assembly of MTGs-containing repressor complexes at specific target sites in the genome remains to be established. A growing number of RNAs, including non-coding RNAs, appears to be implicated in chromatin architecture and chromatin-mediated transcriptional regulation [28-30]. Due to the already known networking ability of the MTG proteins [20], it is possible that these family of proteins are even more versatile than originally expected, being capable of networking regulatory RNA in addition to chromatin regulatory/remodelling complexes at specific sites of the genome.

## Conclusion

Evidence has been accumulating that RNA plays a role in transcriptional control. Both wild type MTGs and leukemia-associated AML1-MTG fusion proteins display novel *in vitro *RNA-binding properties. These findings lend support to the hypothesis of the involvement of an RNA component in MTG-mediated chromatin regulation.

## Methods

### *In silico *analyses

Prediction of RNA-binding residues in the primary structure of the MTG8b protein (Acc. # NP_783552) was performed by using the both the BindN [39] and the RNABindR [43] software. Prediction of the positively charged, nucleid-acid-binding patches of the MTG8 NHR4 solution structure (PDB ID # 2OD1) was performed by using Patch Finder Plus [41,42]. The SBR region was further analyzed by multiple alignments with both the MTG8 proteins of different species (mouse MTG8, Acc. # NP_001104497; Chicken MTG8, Acc. # NP_990075, Xenopus MTG8, Acc. # NP_001089065) and the other human MTG proteins (MTG16a, Acc. # NP_005178; MTGR1b, Acc # NP_005084). Multiple alignments were obtained by using the DNAman software followed by minor manual adjustments.

### Cell cultures and transfections

COS-7 were cultured in Dulbecco's modified Eagle's medium (DMEM) supplemented with 10% fetal calf serum (FCS) and 1% antibiotics (penicillin and streptomycin) at 37°C and 5% CO_2_. Cells were transiently transfected with 1 μg of plasmid DNA and Lipofectamine Plus (Invitrogen, Carlsbad, CA) according to the manufacturer's instructions and harvested after 48 h.

### Constructs

The psf2 construct containing *FMR1 *cDNA was previously described [47]. The cDNAs of wild type MTGs, AML1-MTGs and MTG8 deletion mutants were subcloned by PCR into the CMV-driven mammalian expression vector pcDNA3.1/V5-His TOPO (Invitrogen) leaving or introducing a stop-codon before the V5-His tag. *MTG16a *cDNA was kindly provided by Drs Kosoda and Ohki (National Cancer Research Institute, Tokyo, Japan) and amplified with primers P44 (5'-ACC ATG CCG GCT TCA AGA CT-3') and P10 (5'-CAG GGG CCA GTG GGG TCA-3'). *MTGR1a *cDNA was kindly provided by Dr I. Kitabayashi (National Cancer Research Institute, Tokyo, Japan) and amplified with primers P12 (5'-AAC CAT GCC TGG ATC GCC TG-3') and P13 (5'-AGC AGA GTC CGG GGC TCA G-3'). The cDNA of wild type *MTG8b *was amplified from pCMV-MTG8b [16] with primers P122 (5'-ACC ATG ATA TCT GTC AAA AGA AAC-3') and P7 (5'-TCA CGT CTA GCG AGG GGT TG-3'). The MTG8 deletion mutants were amplified from *MTG8b *cDNA with the following primers: MTG8Δ1, lacking the region coding for amino acids 449–604, with primers P122 and P123 (5'-CTC CTC AGC TTA CTT CCA GAT C-3'); MTG8Δ2, lacking the region coding for amino acids 1–304, with primers P125 (5'-ACC ATG GCC ATT GCC CAC CAC TAC-3') and P7; MTG8Δ3, lacking the region coding for amino acids 1–329, with primers P157 (5'-GTT ATG GGG TTG CAT GGC ACA CG-3') and P7; MTG8Δ4, lacking the region coding for amino acids 1–329 and 514–604, with primers P157 and P124 (5'-CCA GCA ACT CTA GCT TGA ATC C-3'). The cDNAs of *AML1-MTG8 *and *AML1-MTG16 *type1 were kindly provided by Dr I. Kitabayashi and amplified with primers P51 (5'-ACC ATG CGT ATC CCC GTA GAT G-3') and P7, and P51–P10, respectively.

### *In vitro *transcription translation

*In vitro *transcription/translation was performed with TnT Quick Coupled Transcription/Translation System (Promega, Madison, WI) according to the manufacturer's instructions. 1 μg of plasmid DNA containing a T7 promoter was used in each reaction. The samples were directly used for the RNA-binding assay.

### RNA-binding assay

The RNA-binding assay was based on the affinity for different RNA homopolymers, as previously described [32]. Approximately 10^6 ^transfected cells were homogenized in binding buffer (10 mM Tris-HCl pH 7.5, 2.5 mM MgCl_2_, 0.5% Triton X100) containing NaCl in the indicated concentrations (150 mM when not mentioned otherwise) and 50 μM ZnCl_2 _when not otherwise indicated. The samples were sonicated 2–3 times for 15 sec. and spun down for 5' at 13000 rpm at 4°C. A small part of the homogenate was saved as total sample and the rest was split and used for binding to 60 μl of Sepharose 4B-poly(U), -poly(A) (both from GE Healthcare, Piscataway, NJ), -poly (C) or -poly(G) (both from Sigma) beads previously washed and diluted 1:1 in binding buffer. As a negative control we either used beads treated with 50 U/ml micrococcal nuclease for 1 h 30°C or ECH Sepharose 4B (GE Healthcare). After incubation at 4°C for 1 h on a rocking platform, the beads were washed 5 times with 1 ml binding buffer and finally eluted with NuPage LDS Sample Buffer (Invitrogen). Samples were analyzed on SDS-PAGE followed by Western blotting. Immunodetection was performed with antibodies that we developed against MTG8 (AB-8, rabbit polyclonal, 1:2000), MTG16 (AB-16, rabbit polyclonal, 1:2000), MTGR1 (AB-R1, rabbit polyclonal, 1:2000) [16], and FMRP (1A, mouse monoclonal, 1:4000) [35]. Commercial antibodies were used for BSA (Sigma, mouse monoclonal, 1:1000) and GFP (Roche, mouse monoclonal, 1:1000). Incubation with the primary antibody was followed by incubation with HRP-conjugated anti-rabbit and anti-mouse secondary antibodies (GE Healthcare, 1:5000) and ECL detection (GE Healthcare).

## Authors' contributions

SR contributed to conceive the hypothesis, performed the *in silico *analyses, designed and carried out the experiments, and drafted the manuscript. LV contributed to perform the RNA-binding experiments. NS contributed to conceive the hypothesis, co-ordinated the overall project, and critically revised the manuscript. ATH contributed to conceive the hypothesis, provided experimental advice, and reviewed the manuscript.
